# Methyl (*Z*)-2-{[*N*-(2-formyl­phen­yl)-4-methyl­benzene­sulfonamido]­meth­yl}-3-phenyl­prop-2-enoate

**DOI:** 10.1107/S1600536811050756

**Published:** 2011-11-30

**Authors:** R. Madhanraj, S. Murugavel, D. Kannan, M. Bakthadoss

**Affiliations:** a Department of Physics, Ranipettai Engineering College, Thenkadapathangal, Walaja 632 513, India; b Department of Physics, Thanthai Periyar Government Institute of Technology, Vellore 632 002, India; c Department of Organic Chemistry, University of Madras, Maraimalai Campus, Chennai 600 025, India

## Abstract

In the title compound, C_25_H_23_NO_5_S, the sulfonyl-bound benzene ring forms dihedral angles of 37.2 (1) and 67.0 (1)°, respectively, with the formyl­phenyl and phenyl rings. The mol­ecular conformation is stabilized by an intra­molecular C—H⋯π inter­action. In the crystal, mol­ecules are linked by C—H⋯O hydrogen bonds, forming a two-dimensional network in the (110) plane in which *R*_4_^4^(38) ring motifs are generated.

## Related literature

For background to the pharmacological uses of sulfonamides, see: Korolkovas (1988 ▶); Mandell & Sande (1992 ▶). For related structures, see: Ranjith *et al.* (2009 ▶); Aziz-ur-Rehman *et al.* (2010 ▶). For hydrogen-bond motifs, see: Bernstein *et al.* (1995 ▶). For the Thrope–Ingold effect, see: Bassindale (1984 ▶).
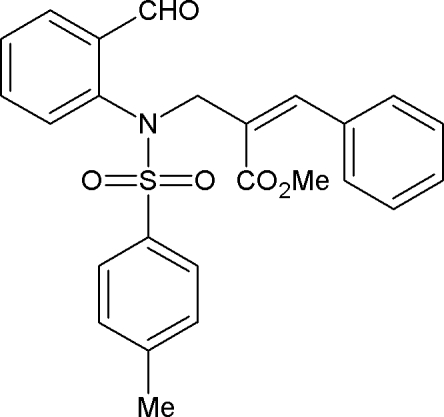


## Experimental

### 

#### Crystal data

C_25_H_23_NO_5_S
*M* *_r_* = 449.50
Monoclinic, 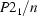

*a* = 9.7475 (5) Å
*b* = 21.7053 (12) Å
*c* = 11.2643 (6) Å
β = 109.987 (2)°
*V* = 2239.7 (2) Å^3^
*Z* = 4
Mo *K*α radiation
μ = 0.18 mm^−1^
*T* = 293 K
0.23 × 0.21 × 0.16 mm

#### Data collection

Bruker APEXII CCD diffractometer
Absorption correction: multi-scan (*SADABS*; Sheldrick, 1996 ▶) *T*_min_ = 0.959, *T*_max_ = 0.971
28975 measured reflections
6991 independent reflections
4593 reflections with *I* > 2σ(*I*)
*R*_int_ = 0.028

#### Refinement

*R*[*F* ^2^ > 2σ(*F* ^2^)] = 0.048
*wR*(*F* ^2^) = 0.153
*S* = 0.99
6991 reflections
291 parameters
H-atom parameters constrained
Δρ_max_ = 0.45 e Å^−3^
Δρ_min_ = −0.31 e Å^−3^

### 

Data collection: *APEX2* (Bruker, 2004 ▶); cell refinement: *APEX2* and *SAINT* (Bruker, 2004 ▶); data reduction: *SAINT* and *XPREP* (Bruker, 2004 ▶); program(s) used to solve structure: *SHELXS97* (Sheldrick, 2008 ▶); program(s) used to refine structure: *SHELXL97* (Sheldrick, 2008 ▶); molecular graphics: *ORTEP-3* (Farrugia (1997 ▶); software used to prepare material for publication: *SHELXL97* and *PLATON* (Spek, 2009 ▶).

## Supplementary Material

Crystal structure: contains datablock(s) global, I. DOI: 10.1107/S1600536811050756/bt5721sup1.cif

Structure factors: contains datablock(s) I. DOI: 10.1107/S1600536811050756/bt5721Isup2.hkl

Supplementary material file. DOI: 10.1107/S1600536811050756/bt5721Isup3.cml

Additional supplementary materials:  crystallographic information; 3D view; checkCIF report

## Figures and Tables

**Table 1:** Hydrogen-bond geometry (Å, °) *Cg* is the centroid of the C18–C23 ring.

*D*—H⋯*A*	*D*—H	H⋯*A*	*D*⋯*A*	*D*—H⋯*A*
C9—H9⋯*Cg*	0.93	2.64	3.470 (2)	149
C25—H25*B*⋯O2^i^	0.96	2.56	3.342 (3)	139
C10—H10⋯O1^ii^	0.93	2.51	3.309 (3)	145

Symmetry codes: (i) 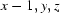; (ii) 
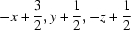
.

**Fig. 1.**
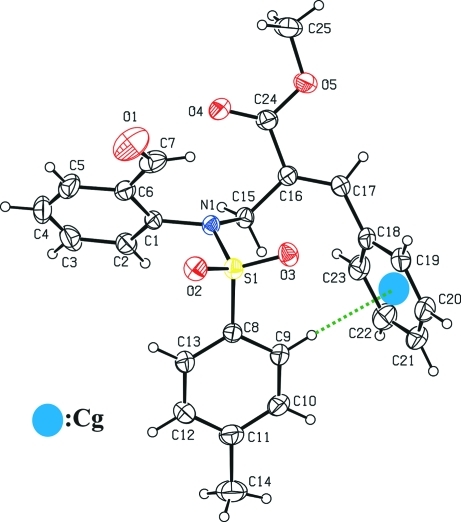
The molecular structure of the title compound with intramolecular C—H···π interactions shown as dashed lines. Displacement ellipsoids are drawn at the 30% probability levels. H atoms are presented as a small spheres of arbitrary radius. Cg is the centroid of the C18–C23 ring.

**Fig. 2.**
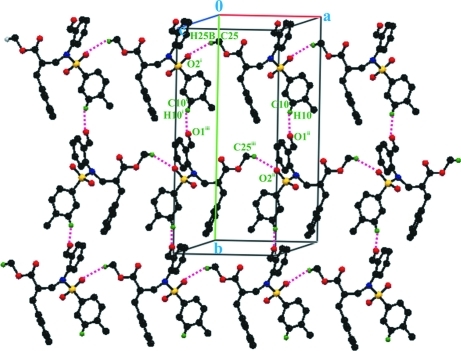
Part of the crystal structure showing C—H···O hydrogen bonds (dotted lines) , with R44(38) ring motifs. [Symmetry code: (i) -1+x, y, z; (ii) 3/2-x, 1/2+y, 1/2-z; (iii) 1/2-x, 1/2+y, 1/2-z].

## supplementary crystallographic information

### Comment

Sulfonamide drugs are widely used for the treatment of certain infections caused by Gram-positive and Gram-negative microorganisms, some fungi, and certain protozoa (Korolkovas, 1988, Mandell & Sande, 1992). In view of this biological importance, the crystal structure of the title compound has been determined and the results are presented here.

Fig. 1. shows a displacement ellipsoid plot of (I), with the atom numbering scheme. The S1 atom shows a distorted tetrahedral geometry, with O2—S1—O3 [119.6 (1)°] and N1—S1—C8 [107.5 (1)°] angles deviating from ideal tetrahedral values, are attributed to the Thrope-Ingold effect (Bassindale, 1984). The sum of bond angles around N1 (351.5°) indicates that N1 is in *sp*^2^ hybridization. The sulfonyl bound phenyl (C8–C13) ring forms dihedral angles of 37.2 (1)° and 67.0 (1)°, respectively, with the formyl phenyl (C1–C6) and phenyl (C18—C23) rings. The dihedral angle between formyl phenyl and phenyl rings is 45.9 (1)°. The geometric parameters of the title molecule agrees well with those reported for similar structures (Ranjith *et al.,* 2009; Aziz-ur-Rehman *et al.,* 2010).

The molecular structure is stabilized by intramolecular C-H···π interaction between a sulfonyl bound phenyl H9 atom and a phenyl (C18–C23) ring with a C9—H9···Cg seperation of 2.64 Å.(Fig. 1 and Table 1; Cg is the centroid of the C18–C23 phenyl ring). In the crystal four molecules are linked by intermolecular C—H···O hydrogen bonds (Fig. 2, Table 1; Symmetry codes as given in Fig. 2), generating *R_4_^4^*(38) ring motifs (Bernstein *et al.,* 1995) to form a two dimensional network along [110]] directions.

### Experimental

A solution of *N*-(formylphenyl)(4-methylbenzene)sulfonamide (1 mmol, 0.28 g) and potassium carbonate (1.5 mmol, 0.21 g) in acetonitrile solvent was stirred for 15 minutes at room temperature. To this solution, (*z*)-methyl-2-(bromomethyl)-3-phenylprop-2-enoate (1.2 mmol, 0.30 g) was added dropwise till the addition is complete. After the completion of the reaction, as indicated by TLC, acetonitile was evaporated. ETOAc (15 ml) were added to the crude mass. The organic layer was dried over anhydrous sodium sulfate. Removal of solvent led to the crude product, which was purified through pad of silica gel (100-200 mesh) using ethylacetate and hexanes (1:9) as solvents. The pure title compound was obtained as a colourless solid (0.40 g, 88 % yield). Recrystallization was carried out using ethylacetate as solvent.

### Refinement

All the H atoms were positioned geometrically, with C–H = 0.93–0.97 Å and constrained to ride on their parent atom, with *U*_iso_(H) =1.5*U*_eq_ for methyl H atoms and 1.2*U*_eq_(C) for other H atoms.

### Crystal data

**Table:**

C_25_H_23_NO_5_S	*F*(000) = 944
*M**_r_* = 449.50	*D*_x_ = 1.333 Mg m^−^^3^
Monoclinic, *P*2_1_/*n*	Mo *K*α radiation, λ = 0.71073 Å
Hall symbol: -P 2yn	Cell parameters from 7038 reflections
*a* = 9.7475 (5) Å	θ = 2.1–30.8°
*b* = 21.7053 (12) Å	µ = 0.18 mm^−^^1^
*c* = 11.2643 (6) Å	*T* = 293 K
β = 109.987 (2)°	Block, colourless
*V* = 2239.7 (2) Å^3^	0.23 × 0.21 × 0.16 mm
*Z* = 4	

### Data collection

**Table:**

Bruker APEXII CCD diffractometer	6991 independent reflections
Radiation source: fine-focus sealed tube	4593 reflections with *I* > 2σ(*I*)
graphite	*R*_int_ = 0.028
Detector resolution: 10.0 pixels mm^-1^	θ_max_ = 30.8°, θ_min_ = 2.1°
ω scans	*h* = −13→14
Absorption correction: multi-scan (*SADABS*; Sheldrick, 1996)	*k* = −31→31
*T*_min_ = 0.959, *T*_max_ = 0.971	*l* = −16→15
28975 measured reflections	

### Refinement

**Table:**

Refinement on *F*^2^	Primary atom site location: structure-invariant direct methods
Least-squares matrix: full	Secondary atom site location: difference Fourier map
*R*[*F*^2^ > 2σ(*F*^2^)] = 0.048	Hydrogen site location: inferred from neighbouring sites
*wR*(*F*^2^) = 0.153	H-atom parameters constrained
*S* = 0.99	*w* = 1/[σ^2^(*F*_o_^2^) + (0.0773*P*)^2^ + 0.5464*P*] where *P* = (*F*_o_^2^ + 2*F*_c_^2^)/3
6991 reflections	(Δ/σ)_max_ < 0.001
291 parameters	Δρ_max_ = 0.45 e Å^−^^3^
0 restraints	Δρ_min_ = −0.31 e Å^−^^3^

### Special details

**Table:**

Geometry. All esds (except the esd in the dihedral angle between two l.s. planes) are estimated using the full covariance matrix. The cell esds are taken into account individually in the estimation of esds in distances, angles and torsion angles; correlations between esds in cell parameters are only used when they are defined by crystal symmetry. An approximate (isotropic) treatment of cell esds is used for estimating esds involving l.s. planes.
Refinement. Refinement of F^2^ against ALL reflections. The weighted R-factor wR and goodness of fit S are based on F^2^, conventional R-factors R are based on F, with F set to zero for negative F^2^. The threshold expression of F^2^ > 2sigma(F^2^) is used only for calculating R-factors(gt) etc. and is not relevant to the choice of reflections for refinement. R-factors based on F^2^ are statistically about twice as large as those based on F, and R- factors based on ALL data will be even larger.

### Fractional atomic coordinates and isotropic or equivalent isotropic displacement parameters (Å^2^)

**Table:**

	*x*	*y*	*z*	*U*_iso_*/*U*_eq_	
C1	0.59422 (16)	0.13486 (7)	0.03835 (16)	0.0388 (3)	
C2	0.58668 (19)	0.14617 (9)	−0.08453 (18)	0.0511 (4)	
H2	0.5590	0.1848	−0.1206	0.061*	
C3	0.6208 (2)	0.09931 (12)	−0.1534 (2)	0.0703 (6)	
H3	0.6153	0.1067	−0.2362	0.084*	
C4	0.6628 (2)	0.04221 (12)	−0.1010 (3)	0.0785 (8)	
H4	0.6865	0.0113	−0.1478	0.094*	
C5	0.6695 (2)	0.03105 (9)	0.0194 (3)	0.0689 (6)	
H5	0.6980	−0.0077	0.0545	0.083*	
C6	0.63450 (18)	0.07667 (8)	0.09133 (19)	0.0485 (4)	
C7	0.6342 (2)	0.06179 (9)	0.2188 (2)	0.0643 (5)	
H7	0.5862	0.0886	0.2558	0.077*	
C8	0.76439 (16)	0.27383 (7)	0.14360 (16)	0.0407 (3)	
C9	0.7236 (2)	0.33478 (8)	0.1488 (2)	0.0536 (4)	
H9	0.6608	0.3452	0.1919	0.064*	
C10	0.7762 (2)	0.37946 (9)	0.0900 (2)	0.0621 (5)	
H10	0.7491	0.4203	0.0942	0.075*	
C11	0.8691 (2)	0.36516 (9)	0.0245 (2)	0.0581 (5)	
C12	0.9085 (2)	0.30403 (10)	0.0192 (2)	0.0580 (5)	
H12	0.9704	0.2937	−0.0248	0.070*	
C13	0.85695 (19)	0.25836 (8)	0.07857 (18)	0.0502 (4)	
H13	0.8843	0.2176	0.0748	0.060*	
C14	0.9305 (3)	0.41529 (12)	−0.0355 (3)	0.0846 (8)	
H14A	0.8850	0.4538	−0.0294	0.127*	
H14B	0.9117	0.4055	−0.1227	0.127*	
H14C	1.0339	0.4184	0.0076	0.127*	
C15	0.42926 (16)	0.22099 (7)	0.05346 (15)	0.0384 (3)	
H15A	0.3777	0.2057	−0.0310	0.046*	
H15B	0.4598	0.2629	0.0459	0.046*	
C16	0.32755 (16)	0.22105 (7)	0.12797 (16)	0.0403 (3)	
C17	0.29963 (18)	0.27004 (8)	0.18717 (17)	0.0463 (4)	
H17	0.2376	0.2639	0.2331	0.056*	
C18	0.35677 (19)	0.33301 (8)	0.18761 (18)	0.0467 (4)	
C19	0.4342 (2)	0.36162 (9)	0.3013 (2)	0.0568 (5)	
H19	0.4488	0.3411	0.3771	0.068*	
C20	0.4895 (2)	0.42009 (11)	0.3023 (3)	0.0698 (6)	
H20	0.5435	0.4384	0.3786	0.084*	
C21	0.4654 (3)	0.45140 (10)	0.1912 (3)	0.0723 (6)	
H21	0.5030	0.4909	0.1923	0.087*	
C22	0.3860 (3)	0.42452 (10)	0.0785 (3)	0.0717 (6)	
H22	0.3679	0.4461	0.0034	0.086*	
C23	0.3326 (2)	0.36523 (9)	0.0762 (2)	0.0581 (5)	
H23	0.2801	0.3470	−0.0006	0.070*	
C24	0.25669 (18)	0.16079 (8)	0.13063 (19)	0.0492 (4)	
C25	0.0837 (3)	0.10598 (10)	0.1935 (3)	0.0819 (8)	
H25A	0.0173	0.0964	0.1106	0.123*	
H25B	0.0302	0.1106	0.2504	0.123*	
H25C	0.1534	0.0732	0.2225	0.123*	
N1	0.56062 (13)	0.18221 (6)	0.11335 (12)	0.0362 (3)	
O1	0.6918 (3)	0.01735 (8)	0.2780 (2)	0.1093 (7)	
O2	0.81113 (13)	0.17074 (6)	0.26567 (12)	0.0525 (3)	
O3	0.64184 (13)	0.24539 (6)	0.30692 (11)	0.0509 (3)	
O4	0.28273 (17)	0.11474 (6)	0.08321 (18)	0.0714 (4)	
O5	0.15907 (17)	0.16258 (6)	0.18906 (17)	0.0694 (4)	
S1	0.69945 (4)	0.216238 (18)	0.22055 (4)	0.04001 (12)	

### Atomic displacement parameters (Å^2^)

**Table:**

	*U*^11^	*U*^22^	*U*^33^	*U*^12^	*U*^13^	*U*^23^
C1	0.0321 (7)	0.0374 (7)	0.0488 (9)	0.0001 (5)	0.0164 (6)	−0.0058 (6)
C2	0.0459 (9)	0.0582 (10)	0.0512 (10)	−0.0033 (8)	0.0193 (8)	−0.0072 (8)
C3	0.0544 (11)	0.0992 (18)	0.0651 (13)	−0.0141 (11)	0.0303 (10)	−0.0337 (12)
C4	0.0581 (12)	0.0746 (15)	0.110 (2)	−0.0031 (11)	0.0379 (13)	−0.0465 (15)
C5	0.0499 (11)	0.0440 (10)	0.111 (2)	0.0040 (8)	0.0246 (11)	−0.0185 (11)
C6	0.0378 (8)	0.0379 (8)	0.0690 (12)	0.0009 (6)	0.0171 (8)	−0.0013 (8)
C7	0.0626 (12)	0.0452 (10)	0.0788 (14)	−0.0004 (9)	0.0162 (10)	0.0150 (9)
C8	0.0335 (7)	0.0407 (8)	0.0468 (9)	−0.0019 (6)	0.0123 (6)	−0.0067 (7)
C9	0.0521 (10)	0.0427 (9)	0.0728 (13)	−0.0015 (7)	0.0303 (9)	−0.0119 (8)
C10	0.0616 (12)	0.0396 (9)	0.0895 (16)	−0.0035 (8)	0.0315 (11)	−0.0069 (9)
C11	0.0521 (10)	0.0551 (11)	0.0669 (12)	−0.0117 (8)	0.0202 (9)	−0.0023 (9)
C12	0.0527 (10)	0.0645 (12)	0.0647 (12)	−0.0048 (9)	0.0303 (9)	−0.0067 (10)
C13	0.0466 (9)	0.0455 (9)	0.0621 (11)	0.0028 (7)	0.0232 (8)	−0.0065 (8)
C14	0.0802 (16)	0.0734 (15)	0.108 (2)	−0.0179 (13)	0.0418 (15)	0.0120 (14)
C15	0.0348 (7)	0.0372 (7)	0.0409 (8)	0.0073 (6)	0.0099 (6)	0.0012 (6)
C16	0.0320 (7)	0.0384 (8)	0.0479 (9)	0.0037 (6)	0.0106 (6)	0.0003 (6)
C17	0.0413 (8)	0.0454 (9)	0.0563 (10)	0.0028 (7)	0.0220 (7)	−0.0032 (7)
C18	0.0441 (9)	0.0404 (8)	0.0592 (11)	0.0060 (6)	0.0224 (8)	−0.0079 (7)
C19	0.0601 (11)	0.0573 (11)	0.0589 (11)	−0.0005 (9)	0.0280 (9)	−0.0122 (9)
C20	0.0659 (13)	0.0616 (13)	0.0867 (17)	−0.0097 (10)	0.0322 (12)	−0.0305 (12)
C21	0.0747 (14)	0.0401 (10)	0.112 (2)	−0.0009 (9)	0.0454 (14)	−0.0107 (12)
C22	0.0849 (16)	0.0465 (11)	0.0871 (16)	0.0119 (10)	0.0338 (13)	0.0097 (11)
C23	0.0604 (11)	0.0470 (10)	0.0646 (12)	0.0090 (8)	0.0186 (9)	−0.0022 (9)
C24	0.0366 (8)	0.0423 (9)	0.0684 (12)	0.0030 (6)	0.0176 (8)	0.0034 (8)
C25	0.0644 (14)	0.0548 (12)	0.143 (2)	−0.0024 (10)	0.0567 (15)	0.0158 (14)
N1	0.0321 (6)	0.0343 (6)	0.0406 (7)	0.0043 (5)	0.0102 (5)	−0.0014 (5)
O1	0.1367 (18)	0.0598 (10)	0.1075 (15)	0.0136 (11)	0.0111 (13)	0.0356 (10)
O2	0.0400 (6)	0.0564 (7)	0.0532 (7)	0.0107 (5)	0.0056 (5)	0.0064 (6)
O3	0.0482 (7)	0.0638 (8)	0.0410 (6)	−0.0001 (6)	0.0154 (5)	−0.0111 (6)
O4	0.0677 (9)	0.0446 (7)	0.1143 (13)	−0.0088 (6)	0.0471 (9)	−0.0177 (8)
O5	0.0643 (9)	0.0469 (7)	0.1163 (13)	0.0025 (6)	0.0558 (9)	0.0068 (8)
S1	0.03449 (19)	0.0436 (2)	0.0391 (2)	0.00304 (14)	0.00894 (15)	−0.00255 (15)

### Geometric parameters (Å, °)

**Table:**

C1—C2	1.383 (3)	C15—N1	1.4875 (18)
C1—C6	1.395 (2)	C15—C16	1.502 (2)
C1—N1	1.4375 (19)	C15—H15A	0.9700
C2—C3	1.387 (3)	C15—H15B	0.9700
C2—H2	0.9300	C16—C17	1.332 (2)
C3—C4	1.374 (4)	C16—C24	1.484 (2)
C3—H3	0.9300	C17—C18	1.475 (2)
C4—C5	1.357 (4)	C17—H17	0.9300
C4—H4	0.9300	C18—C23	1.385 (3)
C5—C6	1.394 (3)	C18—C19	1.391 (3)
C5—H5	0.9300	C19—C20	1.377 (3)
C6—C7	1.473 (3)	C19—H19	0.9300
C7—O1	1.197 (2)	C20—C21	1.372 (4)
C7—H7	0.9300	C20—H20	0.9300
C8—C13	1.384 (2)	C21—C22	1.371 (4)
C8—C9	1.388 (2)	C21—H21	0.9300
C8—S1	1.7577 (17)	C22—C23	1.385 (3)
C9—C10	1.369 (3)	C22—H22	0.9300
C9—H9	0.9300	C23—H23	0.9300
C10—C11	1.385 (3)	C24—O4	1.201 (2)
C10—H10	0.9300	C24—O5	1.330 (2)
C11—C12	1.388 (3)	C25—O5	1.441 (2)
C11—C14	1.508 (3)	C25—H25A	0.9600
C12—C13	1.383 (3)	C25—H25B	0.9600
C12—H12	0.9300	C25—H25C	0.9600
C13—H13	0.9300	N1—S1	1.6485 (13)
C14—H14A	0.9600	O2—S1	1.4284 (12)
C14—H14B	0.9600	O3—S1	1.4268 (12)
C14—H14C	0.9600		
			
C2—C1—C6	119.99 (16)	C16—C15—H15A	109.2
C2—C1—N1	121.11 (15)	N1—C15—H15B	109.2
C6—C1—N1	118.90 (15)	C16—C15—H15B	109.2
C1—C2—C3	119.3 (2)	H15A—C15—H15B	107.9
C1—C2—H2	120.4	C17—C16—C24	121.16 (16)
C3—C2—H2	120.4	C17—C16—C15	124.56 (15)
C4—C3—C2	121.0 (2)	C24—C16—C15	114.28 (14)
C4—C3—H3	119.5	C16—C17—C18	126.87 (16)
C2—C3—H3	119.5	C16—C17—H17	116.6
C5—C4—C3	119.7 (2)	C18—C17—H17	116.6
C5—C4—H4	120.1	C23—C18—C19	118.66 (18)
C3—C4—H4	120.1	C23—C18—C17	121.18 (17)
C4—C5—C6	121.1 (2)	C19—C18—C17	120.15 (18)
C4—C5—H5	119.5	C20—C19—C18	120.5 (2)
C6—C5—H5	119.5	C20—C19—H19	119.8
C5—C6—C1	118.9 (2)	C18—C19—H19	119.8
C5—C6—C7	119.56 (19)	C21—C20—C19	120.3 (2)
C1—C6—C7	121.45 (17)	C21—C20—H20	119.9
O1—C7—C6	124.2 (2)	C19—C20—H20	119.9
O1—C7—H7	117.9	C22—C21—C20	120.0 (2)
C6—C7—H7	117.9	C22—C21—H21	120.0
C13—C8—C9	120.10 (17)	C20—C21—H21	120.0
C13—C8—S1	119.88 (13)	C21—C22—C23	120.2 (2)
C9—C8—S1	120.02 (13)	C21—C22—H22	119.9
C10—C9—C8	119.64 (17)	C23—C22—H22	119.9
C10—C9—H9	120.2	C18—C23—C22	120.4 (2)
C8—C9—H9	120.2	C18—C23—H23	119.8
C9—C10—C11	121.36 (18)	C22—C23—H23	119.8
C9—C10—H10	119.3	O4—C24—O5	122.70 (17)
C11—C10—H10	119.3	O4—C24—C16	123.65 (17)
C10—C11—C12	118.55 (19)	O5—C24—C16	113.64 (15)
C10—C11—C14	120.6 (2)	O5—C25—H25A	109.5
C12—C11—C14	120.8 (2)	O5—C25—H25B	109.5
C13—C12—C11	120.84 (18)	H25A—C25—H25B	109.5
C13—C12—H12	119.6	O5—C25—H25C	109.5
C11—C12—H12	119.6	H25A—C25—H25C	109.5
C12—C13—C8	119.51 (17)	H25B—C25—H25C	109.5
C12—C13—H13	120.2	C1—N1—C15	118.14 (12)
C8—C13—H13	120.2	C1—N1—S1	117.12 (10)
C11—C14—H14A	109.5	C15—N1—S1	116.27 (10)
C11—C14—H14B	109.5	C24—O5—C25	116.87 (16)
H14A—C14—H14B	109.5	O3—S1—O2	119.58 (8)
C11—C14—H14C	109.5	O3—S1—N1	106.39 (7)
H14A—C14—H14C	109.5	O2—S1—N1	106.61 (7)
H14B—C14—H14C	109.5	O3—S1—C8	108.23 (8)
N1—C15—C16	112.16 (12)	O2—S1—C8	107.94 (8)
N1—C15—H15A	109.2	N1—S1—C8	107.53 (7)
			
C6—C1—C2—C3	−0.6 (2)	C18—C19—C20—C21	1.7 (3)
N1—C1—C2—C3	179.61 (15)	C19—C20—C21—C22	0.0 (3)
C1—C2—C3—C4	−0.4 (3)	C20—C21—C22—C23	−1.4 (4)
C2—C3—C4—C5	0.7 (3)	C19—C18—C23—C22	0.6 (3)
C3—C4—C5—C6	0.0 (3)	C17—C18—C23—C22	179.45 (18)
C4—C5—C6—C1	−1.0 (3)	C21—C22—C23—C18	1.1 (3)
C4—C5—C6—C7	176.2 (2)	C17—C16—C24—O4	−176.84 (19)
C2—C1—C6—C5	1.3 (2)	C15—C16—C24—O4	3.6 (3)
N1—C1—C6—C5	−178.91 (15)	C17—C16—C24—O5	4.2 (3)
C2—C1—C6—C7	−175.87 (17)	C15—C16—C24—O5	−175.38 (15)
N1—C1—C6—C7	3.9 (2)	C2—C1—N1—C15	44.2 (2)
C5—C6—C7—O1	17.0 (3)	C6—C1—N1—C15	−135.57 (15)
C1—C6—C7—O1	−165.9 (2)	C2—C1—N1—S1	−102.71 (15)
C13—C8—C9—C10	−0.5 (3)	C6—C1—N1—S1	77.50 (16)
S1—C8—C9—C10	179.12 (16)	C16—C15—N1—C1	125.78 (14)
C8—C9—C10—C11	0.4 (3)	C16—C15—N1—S1	−87.01 (14)
C9—C10—C11—C12	0.0 (3)	O4—C24—O5—C25	−0.1 (3)
C9—C10—C11—C14	−177.7 (2)	C16—C24—O5—C25	178.93 (19)
C10—C11—C12—C13	−0.3 (3)	C1—N1—S1—O3	−161.76 (11)
C14—C11—C12—C13	177.3 (2)	C15—N1—S1—O3	50.69 (13)
C11—C12—C13—C8	0.3 (3)	C1—N1—S1—O2	−33.10 (13)
C9—C8—C13—C12	0.1 (3)	C15—N1—S1—O2	179.35 (11)
S1—C8—C13—C12	−179.45 (14)	C1—N1—S1—C8	82.46 (12)
N1—C15—C16—C17	113.35 (17)	C15—N1—S1—C8	−65.09 (12)
N1—C15—C16—C24	−67.10 (17)	C13—C8—S1—O3	164.21 (13)
C24—C16—C17—C18	−177.05 (17)	C9—C8—S1—O3	−15.39 (17)
C15—C16—C17—C18	2.5 (3)	C13—C8—S1—O2	33.45 (16)
C16—C17—C18—C23	58.2 (3)	C9—C8—S1—O2	−146.15 (15)
C16—C17—C18—C19	−123.0 (2)	C13—C8—S1—N1	−81.23 (15)
C23—C18—C19—C20	−2.0 (3)	C9—C8—S1—N1	99.17 (15)
C17—C18—C19—C20	179.14 (17)		

### Hydrogen-bond geometry (Å, °)

**Table:**

Cg is the centroid of the C18–C23 ring.

**Table:**

*D*—H···*A*	*D*—H	H···*A*	*D*···*A*	*D*—H···*A*
C9—H9···Cg	0.93	2.64	3.470 (2)	149.
C25—H25B···O2^i^	0.96	2.56	3.342 (3)	139.
C10—H10···O1^ii^	0.93	2.51	3.309 (3)	145.

Symmetry codes:  (i) *x*−1, *y*, *z*; (ii) −*x*+3/2, *y*+1/2, −*z*+1/2.
